# Fabrication and Drag Reduction Performance of Bionic Surfaces Featuring Staggered Shield Scale Structures

**DOI:** 10.3390/biomimetics11030209

**Published:** 2026-03-14

**Authors:** Xin Gu, Pan Cao, Xiuqin Bai, Yifeng Fu

**Affiliations:** 1 National Key Laboratory of Autonomous Marine Vehicle Technology, Harbin Engineering University, Harbin 150001, China; 2 School of Mechanical Engineering, Yangzhou University, Yangzhou 225127, China; 3 State Key Laboratory of Maritime Technology and Safety, Wuhan University of Technology, Wuhan 430063, China; 4 School of Automotive and Traffic Engineering, Jiangsu University, Zhenjiang 212013, China

**Keywords:** drag reduction, micro-groove, placoid scale structure, laser etching, biomimetic shark skin, CFD simulation

## Abstract

To investigate the drag reduction mechanism of shark skin placoid scales and develop high-efficiency drag-reducing surfaces, this study designed and fabricated a biomimetic shark skin surface featuring staggered microscale groove structures. The fabrication process involved laser etching on silicon wafers to create a placoid microstructure template, followed by polydimethylsiloxane (PDMS) replication to obtain biomimetic shark skin samples. Sedimentation experiments demonstrated that the biomimetic surface significantly reduced settling time compared to a smooth surface, achieving a drag reduction rate of 5.65%. Further computational fluid dynamics (CFD) simulations were conducted to analyze the near-wall flow characteristics around the biomimetic surface. The results revealed that the drag reduction mechanism primarily stems from the effective regulation of near-wall laminar flow by the micro-groove structures: a low-velocity fluid layer formed within the grooves reduces the near-wall velocity gradient, thereby decreasing frictional drag, while stable recirculation zones develop within the grooves, contributing to momentum redistribution and reduced energy dissipation. Additionally, the staggered arrangement of the grooves promotes a smoother pressure distribution along the flow direction, mitigating pressure drag by reducing the pressure differential between windward and leeward surfaces. The experimental and simulation results showed excellent agreement (simulated drag reduction rate: 5.08%), collectively verifying the feasibility and effectiveness of the proposed biomimetic placoid structure in achieving fluid drag reduction.

## 1. Introduction

In the context of global energy crisis and climate change, energy conservation and emission reduction in the transportation sector have become major international focuses [1,2,3]. Maritime transport, as the predominant mode for international trade, carries the vast majority of global trade volume while also consuming substantial amounts of energy [4,5,6]. During ship navigation, fuel consumption used to overcome frictional resistance between the hull surface and water accounts for 60% to 80% of total resistance [5,7,8,9]. This high energy consumption not only drives up operational costs but also exacerbates greenhouse gas emissions. Research shows that a 10% reduction in ship surface frictional resistance could lead to approximately 3.75% energy savings overall [1,7]. Therefore, developing efficient drag reduction technologies holds significant practical importance for reducing energy consumption in ships and underwater vehicles, enhancing operational performance, and promoting sustainable maritime transport.

Against this background, biomimetics offers a new approach for drag reduction technology. Through long-term natural selection and evolution, biological systems have developed highly optimized structures and functions, providing rich inspiration for solving engineering challenges [2,10,11,12]. In particular, aquatic organisms such as fish have evolved low-drag morphological and surface structures as adaptations to high-speed swimming [4,13,14,15,16,17,18,19,20]. Among them, the microstructure of shark skin stands out: its surface is not entirely smooth but covered with orderly arranged microscale rib-like placoid scales. Studies have shown that such non-smooth surface structures can significantly reduce frictional drag in turbulent flows while offering some anti-fouling properties [21]. This discovery challenges the traditional notion that “smoother surfaces lead to lower resistance” and has stimulated extensive research on biomimetic drag-reducing surfaces [22,23].

The placoid scale structure of real shark skin in nature is extremely complex, exhibiting three-dimensional, multifaceted, and highly curved geometric characteristics that vary with shark species and body location. While this complexity endows shark skin with exceptional hydrodynamic performance, it also presents significant challenges for engineering fabrication [5]. Consequently, a reasonable simplification of the real shark denticle structure is necessary, abstracting it into a micro-groove array with staggered arrangement (like the Sharklet microstructure). Given that the core drag reduction mechanism of shark skin primarily stems from the regulation of near-wall turbulent structures by its oriented micro-grooves, rather than from complex geometric details, and considering engineering fabrication perspectives, simplification into regular groove arrays can significantly reduce processing difficulty, improve structural precision and repeatability, and facilitate subsequent parametric studies and practical applications [6,21]. Therefore, the simplified model adopted in this study retains the core drag reduction mechanism of shark skin while considering engineering feasibility, laying a foundation for the in-depth investigation of the structure–property relationship between microstructural parameters and drag reduction performance.

To gain a deeper understanding and replicate the superior performance of shark skin, researchers have conducted multifaceted investigations into its drag reduction mechanisms, structural optimization, and surface fabrication [24,25]. Mechanistic studies indicate that specific surface microstructures can effectively regulate the evolution of the turbulent boundary layer in the near-wall region. Key mechanisms include the reorganization of flow patterns through physical structures, the lifting and anchoring of streamwise vortices, an increase in viscous sublayer thickness, a reduction in mean velocity gradients, and the suppression of turbulent fluctuations, all contributing to decreased wall shear stress [26,27]. Numerical simulation methods, such as large-eddy simulation and Reynolds-averaged turbulence models, have become essential tools for revealing complex flow-field characteristics and predicting drag reduction performance. Their findings, validated against experimental data from wind tunnels and water channels, have gradually refined the relevant theoretical framework [28].

Currently, research on biomimetic shark skin surfaces is advancing from fundamental mechanistic exploration toward engineering applications. In terms of surface fabrication, traditional mechanical processing and mold replication techniques can produce basic structures but face limitations in achieving nanoscale precision, constructing complex three-dimensional morphologies, and enabling large-scale, efficient production [29,30]. Laser etching technology, with its advantages of high precision, non-contact processing, and broad material compatibility, provides a promising technical route for the precise fabrication of biomimetic microstructures [31]. This technique allows precise control over groove geometry and surface topography, facilitating the study of the structure–property relationship between microstructural parameters and drag reduction performance [32,33].

Despite significant progress in biomimetic shark skin drag reduction research—from initial discovery and mechanistic analysis to applied design and fabrication—several challenges remain [34]. Current studies are often limited to single functions or performance under idealized laboratory conditions, with insufficient attention to the practical implementation of multi-scale optimized structures on engineering material surfaces. A key challenge for practical application lies in developing scalable fabrication processes, such as laser etching, that can simultaneously achieve precise microstructural control while meeting requirements for flexibility, durability, and adaptability to diverse operational environments. Addressing these challenges is crucial for transitioning this technology from laboratory demonstration to real-world applications [35,36,37].

To address this engineering need, the present study employs laser etching technology to fabricate biomimetic drag-reducing surfaces with precisely controlled geometric parameters, based on the principles of shark skin microstructure. Through an integrated approach combining systematic experimentation and numerical simulation, we investigate the influence of microstructural parameters on drag reduction performance and analyze the underlying flow mechanisms. This research not only expands the application of laser etching technology in the fabrication of biomimetic functional surfaces but also provides a theoretical foundation for the design of high-performance drag-reducing surfaces for maritime applications.

## 2. Experimental Section

### 2.1. Materials

PDMS (Part A and Part B, Sylgard 184) was obtained from Dow Corning (Midland, MI, USA). Square-shaped monocrystalline high-purity silicon wafers with dimensions of 10 mm × 10 mm × 1 mm were sourced from a commercial supplier. Anhydrous ethanol (≥99.7%) was purchased from Sinopharm Chemical Reagent Co., Ltd. (Shanghai, China). All chemicals were used as received without further purification.

### 2.2. Fabrication of the Biomimetic Surface

**Figure 1:**
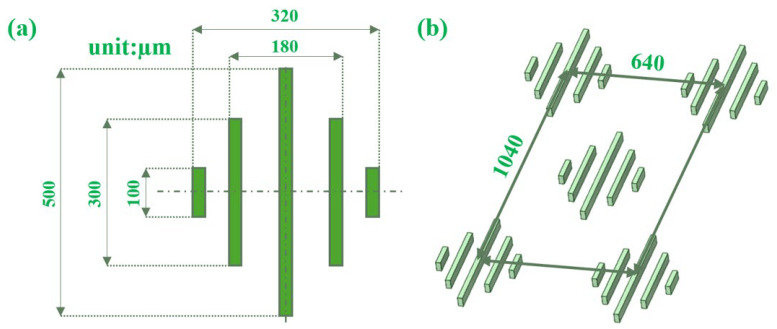
(**a**) Schematic of the simplified placoid scale structure. (**b**) Illustration of the staggered arrangement pattern.

First, the simplified placoid scale geometry was designed using SolidWorks 2024 software, with a single groove width of 20 μm consisting of five ribs (Figure 1a). A staggered arrangement pattern was selected based on previous studies demonstrating its superior drag reduction performance compared to aligned arrangements [21], as depicted in Figure 1b.

**Figure 2:**
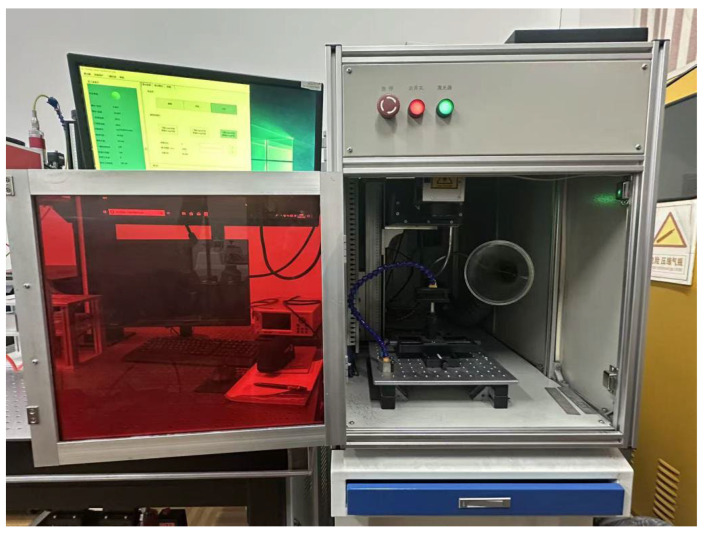
Schematic Diagram of Nanosecond Green Laser.

Subsequently, the silicon wafer was cleaned ultrasonically in ethanol for 10–15 min and dried at room temperature. A nanosecond green laser (λ = 532 nm, LSG10EA/20EB, Huagong Laser Co., Ltd., Wuhan, China, Figure 2) was employed to fabricate the biomimetic structure with the following parameters: repetition number 100, scanning speed 600 mm/s, current 1 A, frequency 80 kHz. The distance between the laser source and sample surface was maintained at approximately 150 mm. After etching, the silicon template was ultrasonically cleaned and dried.

**Figure 3:**
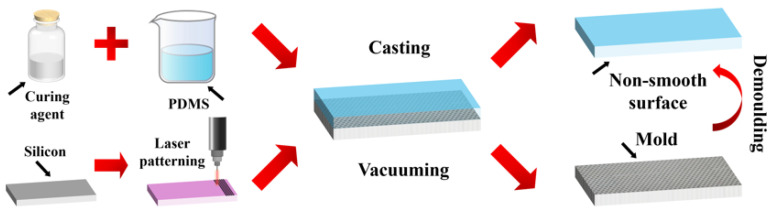
Schematic diagram of the fabrication process for the biomimetic shark skin placoid structure non-smooth surface.

For PDMS replication, the prepolymer and curing agent were mixed at a 10:1 ratio, degassed under vacuum, cast onto the silicon template, and cured at 80 °C for 2 h. The cured PDMS was then carefully demolded to obtain the final biomimetic surface sample (Figure 3). PDMS and curing agent were mixed in a 10:1 ratio under stirring and allowed to degas. The prepared PDMS prepolymer was cast onto the silicon template with the placoid pattern. The casting assembly was placed under vacuum to remove residual bubbles and ensure structural integrity. Subsequently, the PDMS-coated mold was cured at 80 °C for 2 h. After curing, the PDMS was carefully demolded from the silicon template, yielding a non-smooth surface sample with the biomimetic shark skin placoid structure.

### 2.3. Surface Morphology Analysis of Groove Structures

To better evaluate the surface morphology of the processed samples, three-dimensional optical profilometry was employed to perform three-dimensional scanning and measurements on both the laser-etched silicon wafer template surface and the PDMS-demolded non-smooth surface samples. Representative regions featuring the staggered placoid scale arrangement were selected for scanning. Using the accompanying analysis software, key morphological parameters such as surface profile height, groove width, depth, and arrangement spacing were obtained.

**Figure 4:**
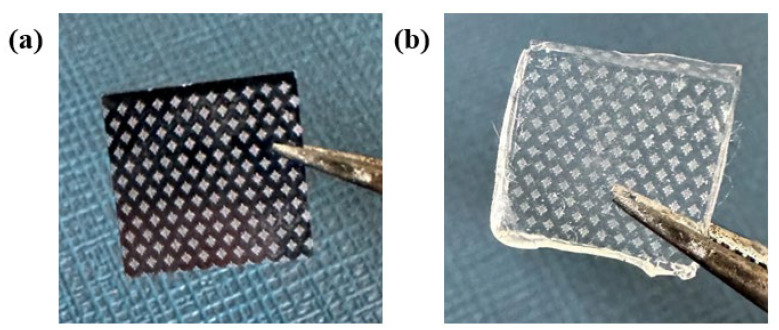
(**a**) Laser-etched template. (**b**) Non-smooth surface sample after PDMS demolding.

**Figure 5:**
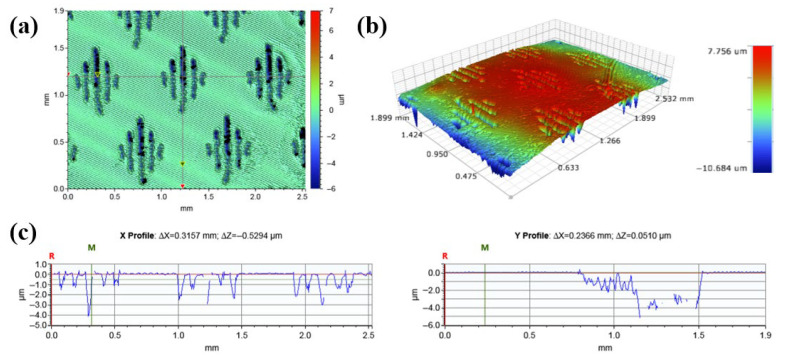
(**a**,**b**) Planar scanning result obtained by 3D profilometer. (**c**) X and Y profile.

The microscopic morphology of the silicon wafer template after nanosecond green laser etching is shown in Figure 4a. It can be observed that the designed simplified placoid groove structure has been clearly transferred onto the silicon surface, with a complete staggered arrangement. The five rib-like groove structures exhibit good continuity and relatively sharp edges, indicating that the selected combination of laser parameters can effectively process the preset microstructure on the silicon substrate. Figure 5a,b displays the three-dimensional morphology and cross-sectional profile analysis of the template surface obtained via 3D optical profilometry. The measurement results show that the average groove width within a single placoid structural unit is (20.5 ± 1.2) μm, which aligns closely with the designed value of 20 μm; the average groove depth is (18.3 ± 0.8) μm. The staggered spacing between adjacent structural units was measured to be (65.4 ± 2.1) μm. The profile curves and X and Y profile further confirm that the grooves possess continuous undulating morphology, as shown in Figure 5c. The transition between the groove bottom and the rib top is relatively smooth, without obvious sharp protrusions or fractures, which is beneficial for subsequent PDMS casting and demolding.

Based on the aforementioned silicon wafer template, an elastomeric surface featuring a positive biomimetic placoid structure was successfully fabricated through the PDMS replication transfer process [21,37]. Figure 4b shows a microscopic image of the PDMS non-smooth surface sample after demolding. It can be seen that the PDMS surface faithfully replicates the staggered placoid array from the template, with continuous structures and no visible defects or residues. Compared to the rigid silicon template, the groove structures on the PDMS sample exhibit slightly rounded edges due to the material’s elasticity. However, the overall morphological features, including groove width, arrangement periodicity, and staggered pattern, are well maintained, demonstrating the feasibility and structural fidelity of the template replication process.

In summary, the morphology analysis results indicate that the laser etching process adopted in this study can precisely fabricate the designed biomimetic placoid micro-groove template. Through the PDMS template replication method, a structurally intact biomimetic non-smooth surface sample was successfully obtained, laying a reliable surface structural foundation for subsequent functional performance tests.

### 2.4. Design of Drag Reduction Experiment

**Figure 6:**
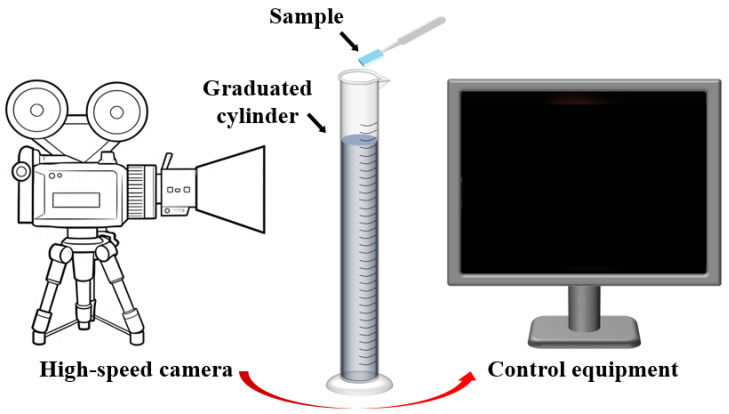
Schematic diagram of the drag reduction experiment.

To quantitatively evaluate the drag reduction performance of the biomimetic shark skin placoid structure, a comparative experiment based on the sedimentation method was designed and conducted in this study. The experimental principle is illustrated in Figure 6. Two types of samples, both with dimensions of 10 mm × 10 mm × 0.1 mm, were selected: one was a smooth-surfaced PDMS sample (control group), and the other was a PDMS sample etched with a biomimetic staggered shark skin placoid structure (experimental group). The experiment was carried out in a transparent glass cylinder with a volume of 500 mL and a height of 40 cm, which was filled with deionized water to provide a stable fluid environment.

During the experiment, each sample was vertically clamped at the same height from the water surface and then released, allowing it to settle freely along the central axis of the cylinder under the influence of gravity. A high-speed camera (frame rate: 500 fps) was used to record the entire process from release until the sample reached the bottom of the cylinder. Displacement–time data were accurately extracted using image processing software. Each type of sample underwent 10 independent experimental repetitions to minimize random errors.

**Figure 7:**
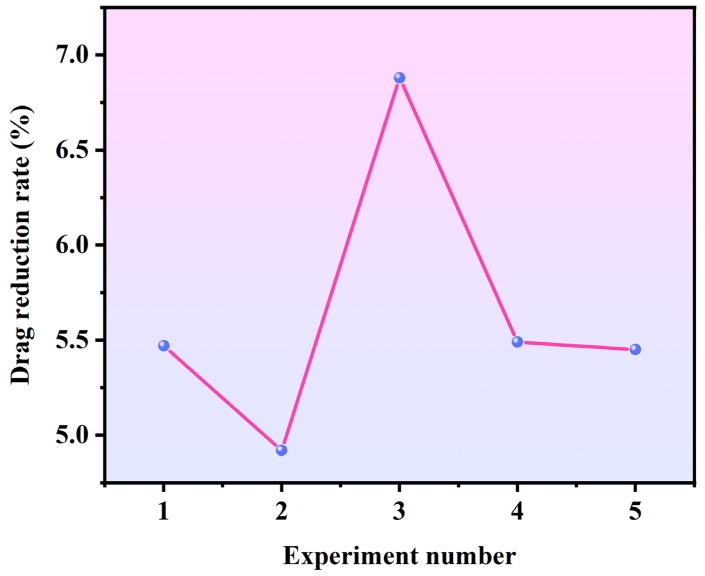
Drag reduction rate obtained for each experimental trial.

Through data analysis of the sedimentation process, the kinematic parameters of the samples were obtained. Table 1 presents the sedimentation time and calculated drag reduction rates from five representative experimental trials. As shown in Figure 7, the drag reduction rates calculated across these five trials are relatively balanced. Based on the height H = 0.4 m, the average sedimentation velocity v and average acceleration a can be calculated using the following formulas:

(1) $$v=\frac{H}{t}$$

(2) $$a=\frac{2H}{t^{2}}$$

The smooth PDMS sample exhibited an average sedimentation time of $t_{\mathrm{smooth}}$ = 2.72 s, corresponding to an average sedimentation velocity of $v_{\mathrm{smooth}}$ = 0.1471 m/s, and an average acceleration of $a_{\mathrm{smooth}}$ = 0.1081 m/s^2^. In contrast, the PDMS sample with biomimetic microstructure demonstrated a reduced average sedimentation time of $t_{\mathrm{bionic}}$ = 2.57 s, with corresponding increases in average velocity and acceleration to $v_{\mathrm{bionic}}$ = 0.1556 m/s and $a_{\mathrm{bionic}}$ = 0.1212 m/s^2^, respectively.

Based on the force analysis of an object settling in a viscous fluid, under conditions where the geometric dimensions, material density, and fluid properties of the samples remain consistent, the difference in sedimentation acceleration can be directly attributed to variations in fluid resistance caused by the surface structure. Accordingly, the drag reduction rate (*DR*) can be estimated using the following formula:

(3) $$DR=\frac{t_{\mathrm{smooth}}-t_{\mathrm{bionic}}}{t_{\mathrm{smooth}}}\times 100\%$$

Substituting the aforementioned average acceleration data, it is calculated that the biomimetic surface achieved an approximate 5.65% drag reduction under the experimental conditions. By rigorously controlling the initial state of the samples (release height, orientation), fluid environment (temperature, purity), and data acquisition methods, this experimental approach effectively ensures the comparability and repeatability of the comparative tests. The quantification of settling time and kinematic parameters provides a concise and effective experimental scenario for intuitively comparing the resistance characteristics of different surfaces, preliminarily validating that the fabricated biomimetic shark skin microstructure possesses significant drag reduction potential in low-speed water flow. Subsequent computational fluid dynamics (CFD) simulations were conducted to further analyze its drag reduction mechanism in depth.

## 3. Numerical Simulation

### 3.1. Geometric Model Construction

To further investigate the drag reduction mechanism of the biomimetic shark skin placoid scale structure and reveal the microscopic details of the near-wall flow field, this chapter employs computational fluid dynamics (CFD) to conduct a three-dimensional numerical simulation of the sample’s sedimentation process in water. The simulation was performed on the commercial software platform ANSYS Fluent 2022 R1. In order to accurately characterize the microstructural features of the biomimetic surface while controlling the computational scale, a computational model containing a periodic structural unit was established in the numerical simulation.

**Figure 8:**
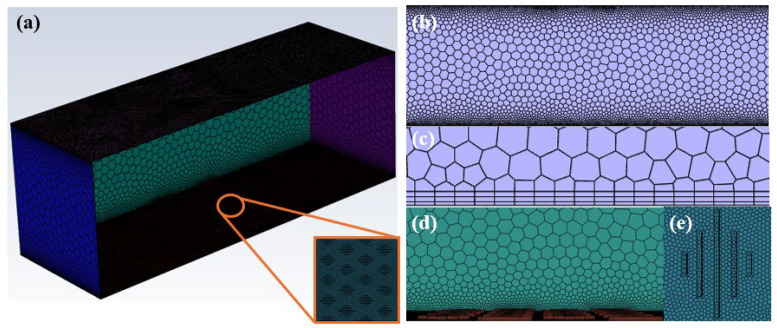
(**a**) Schematic diagram of the biomimetic shark skin placoid structure surface model. (**b**) Mesh distribution adjacent to the wall. (**c**) Refinement of boundary layer meshing. (**d**) Computational domain mesh around the staggered placoid structure. (**e**) Surface mesh discretization of a single shark skin groove structure.

Based on the designed staggered arrangement of the placoid scale structure and its morphological measurement results from the experiments, a simplified three-dimensional geometric model was created at equal scale in SolidWorks. The core of the model is a placoid scale structure representing the basic unit of the staggered array. Its dimensions strictly correspond to the experimental measurements: groove width 20.5 μm, depth 18.3 μm, and unit period 65.4 μm. The model fully reproduces the continuous undulating morphology of the five rib-like grooves, as well as the spatial topology formed by the staggered arrangement, as shown in Figure 8a. To simulate the flow characteristics over an infinitely extended biomimetic surface, periodic boundary conditions were applied in the computational domain setup, extending the basic unit periodically in the streamwise (x-direction) and spanwise (y-direction) [28,38].

### 3.2. Simulation Model Meshing

High-quality mesh generation is critical to ensuring the accuracy and convergence of CFD simulations. Given the complex geometry and microscale features of the biomimetic surface, a multi-region, multi-level refined meshing strategy was adopted in this study, with the optimal mesh scheme determined through mesh independence verification.

To accurately capture the velocity gradient within the near-wall boundary layer, multiple layers of prismatic meshes were applied on the biomimetic surface. As shown in Figure 8b, the meshes adjacent to the biomimetic wall closely conform to the structural contour with smooth transitions. Figure 8c further illustrates the refinement of the boundary layer meshing. Within the laminar model framework, to ensure computational accuracy of the boundary layer velocity distribution, the thickness of the first mesh layer was set to 1 μm. Calculations show that under all simulated flow conditions (0.1–0.2 m/s), the wall y^+^ values are consistently below 1, specifically ranging from 0.1 to 0.3 (depending on flow velocity). This resolution is sufficient to accurately resolve near-wall flow characteristics and meets the mesh requirements for laminar boundary layer solution [38,39,40,41,42].

On this basis, unstructured tetrahedral meshing was applied to the computational domain containing the fundamental unit of the biomimetic structure to flexibly conform to the complex curved geometry. The detailed surface mesh discretization of a single shark skin groove structure is shown in Figure 8e. The mesh size was optimized to balance computational accuracy with computational cost. The overall computational domain mesh, as shown in Figure 8d, exhibits high refinement in the near-wall region, gradually transitioning to coarser meshes toward the core flow area, forming a reasonable mesh gradient distribution. This approach ensured computational accuracy while effectively controlling the total number of mesh elements, resulting in approximately 3.5 million elements in total.

### 3.3. Computational Domain Setup for the Simulation Model

The computational domain was configured as a rectangular prism with the biomimetic structural surface serving as the bottom wall. The domain height (normal direction) was set to be significantly larger than the boundary layer development thickness, to eliminate interference from the upper boundary on the near-wall flow. The lengths in the streamwise (x-direction) and spanwise (y-direction) were set equal to the periodic dimensions of one structural unit, with periodic boundary conditions applied on both sides to simulate fully developed flow over an infinite flat plate [26,39].

Boundary condition setting is a key factor in ensuring the reliability of numerical simulation results. To guarantee the comparability between simulation outcomes and experimental conditions, this study defines the boundaries of the computational domain in detail, with specific configurations provided in Table 2. The upper wall of the computational domain is the smooth surface, while the lower wall is the biomimetic surface with an interleaved shield-scale structure. Both are set as no-slip wall boundary conditions to accurately simulate the constraining effect of solid walls on the fluid. The left and right two side-surfaces are assigned symmetry boundary conditions to emulate the infinite periodic extension of the structure in the spanwise direction. The inlet boundary is configured as a velocity inlet with a speed range of 0.1–0.2 m/s, covering various flow velocity conditions in the experiment and allowing investigation into the dependence of drag reduction performance on flow velocity. The outlet boundary is set as a pressure outlet, with the pressure gradient between the inlet and outlet driving the fluid flow to simulate the sedimentation process observed in the experiment. This boundary configuration comprehensively considers experimental practicality, structural periodicity, and computational stability, laying a solid foundation for subsequent flow field analysis and investigation into drag reduction mechanisms.

To establish a stable and accurate numerical model, systematic configurations for the solution parameters of the computational domain were implemented in this study. Based on the Reynolds number analysis (macroscopic Re ≈ 1000–2000, microscopic Re = 2–13), the flow falls within the laminar to transitional regime; therefore, the laminar model was adopted for numerical simulations [27,40]. The fluid medium was set as water with a density of 998.2 kg/m^3^ and a dynamic viscosity of 1.003 × 10^−3^ Pa·s. The flow velocity within the computational domain was defined in the range of 0.1–0.2 m/s to correspond to the experimental conditions of the sedimentation tests. A pressure-based segregated solver was employed, and the pressure–velocity coupling was handled using the SIMPLEC algorithm to enhance convergence efficiency. Spatial discretization was performed using the second-order upwind scheme to ensure computational accuracy. The convergence criterion was set such that the residuals of all equations fell below 1 × 10^−4^, while key parameters such as wall shear stress were monitored to ensure stability. The maximum number of iterations was set to 1000 to guarantee full convergence of the calculations. The detailed solution parameter settings are summarized in Table 3.

### 3.4. Comparative Analysis of Results

**Figure 9:**
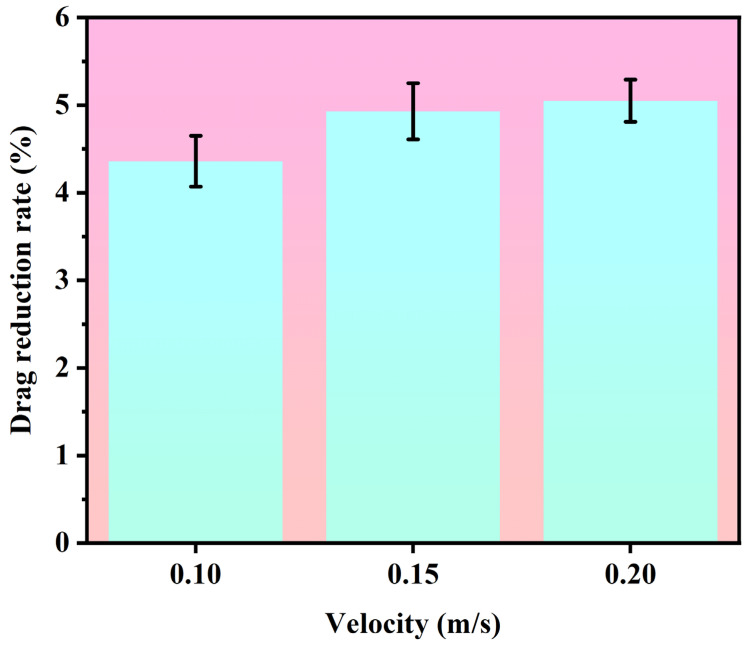
Drag reduction rates obtained at different flow velocities.

To systematically validate and gain deeper insight into the drag reduction performance of the biomimetic shark skin placoid structure, this section presents a comprehensive comparison and mechanistic analysis of experimental observations and numerical simulations. The experimental measurements were complemented by a parametric CFD study investigating the influence of flow velocity and boundary conditions on drag reduction effectiveness, with detailed results compiled in Table 4. The simulation results under no-slip boundary conditions align well with the experimentally observed drag reduction trend. At the characteristic velocity of 0.2 m/s, the simulated drag reduction rate reached 5.08%, closely matching the experimental value of 5.65% derived from settling time measurements. This consistency, with a relative deviation of only 10%, not only validates the accuracy of the experimental setup but also confirms the reliability of the CFD model in capturing the essential flow physics associated with the biomimetic surface. Furthermore, the simulations revealed a distinct non-monotonic relationship between drag reduction and flow velocity under no-slip conditions, as illustrated in Figure 9. The drag reduction rate initially increased with velocity before declining, suggesting that the effectiveness of the micro-groove structure is highly sensitive to flow conditions, possibly due to changes in boundary layer development and vortex interaction mechanisms within the grooves.

A particularly insightful finding emerged from comparing no-slip and free-slip boundary conditions. While the no-slip simulations consistently showed positive drag reduction, the free-slip cases yielded negative values across all tested velocities. This stark contrast underscores the critical role of wall boundary conditions and confirms that the drag reduction mechanism is fundamentally tied to the modification of near-wall flow dynamics—specifically through the development of stable low-velocity fluid layers within the micro-grooves and the formation of recirculation zones that effectively reduce direct fluid–solid interaction. This supports the hypothesis that the structure acts primarily through friction drag modulation rather than through large-scale pressure redistribution. Further decomposition of the drag components in the simulation clarified the underlying physical mechanism in the laminar regime. Under the tested low-speed conditions, the reduction in total drag was predominantly attributable to a decrease in friction drag, which contributed approximately 70–85% of the overall drag reduction depending on velocity. This reduction stems from the lowered near-wall velocity gradient caused by the low-velocity fluid layer anchored within the grooves. Pressure drag, while also influenced by the surface topology, played a secondary role. This aligns with the observed flow fields, where the grooves promote stable recirculation patterns that reduce momentum exchange in the wall-normal direction, thereby lowering shear stress.

The minor discrepancies between the experimental and numerical results can be attributed to several factors. First, unavoidable perturbations during the experimental release process—such as slight rotational motions or off-axis settling—may introduce variability not captured in the idealized CFD model. Second, the physical samples with finite dimensions (10 mm × 10 mm) exhibit three-dimensional edge effects that are not fully represented in the CFD model, which assumes infinite periodic extension. Third, the inherent flexibility of the PDMS material (elastic modulus ≈ 1.8 MPa) may allow slight deformation of the micro-grooves under hydrodynamic loading, resulting in deviations between the effective geometry and the rigid, perfectly defined surfaces employed in the simulation. Fourth, minor variations between the actual manufactured surface morphology and the idealized numerical geometry may also contribute to the observed relative deviation. Under the average flow velocity range of 0.1–0.2 m/s corresponding to the sedimentation experiments, the experimentally measured drag reduction rate was 5.65%, while the maximum drag reduction rate obtained from simulations based on total frictional resistance was 5.08%. Considering the combined influence of the aforementioned factors, this deviation falls within an acceptable range. It is worth noting that the simulation results exhibit an increasing trend in drag reduction rate with flow velocity, which aligns with the physical behavior of drag reduction effectiveness varying with flow velocity observed in experiments, further confirming the reliability of the simulation results. The good consistency between experiment and simulation in terms of drag reduction trends validates the robustness of the observed phenomena.

To clarify the flow regime, the Reynolds number was calculated in this study. It should be noted that the Reynolds number calculation involves two different characteristic length scales: based on the macroscopic sample characteristic length, taking the sample length of 10 mm as the characteristic length, the Reynolds number corresponding to the experimental flow velocity range of 0.1–0.2 m/s is approximately 1000–2000, which corresponds to the laminar to transitional flow regime and is consistent with the laminar model and experimental conditions adopted in this study; based on the microstructural characteristic length, taking the groove width of 20 μm or period of 65 μm as the characteristic length, the corresponding Reynolds number under the same flow velocity is 2–13, which falls completely within the laminar regime. This scale of the Reynolds number reflects the local flow characteristics within the microstructure and differs by orders of magnitude from the macroscopic flow Reynolds number.

**Figure 10:**
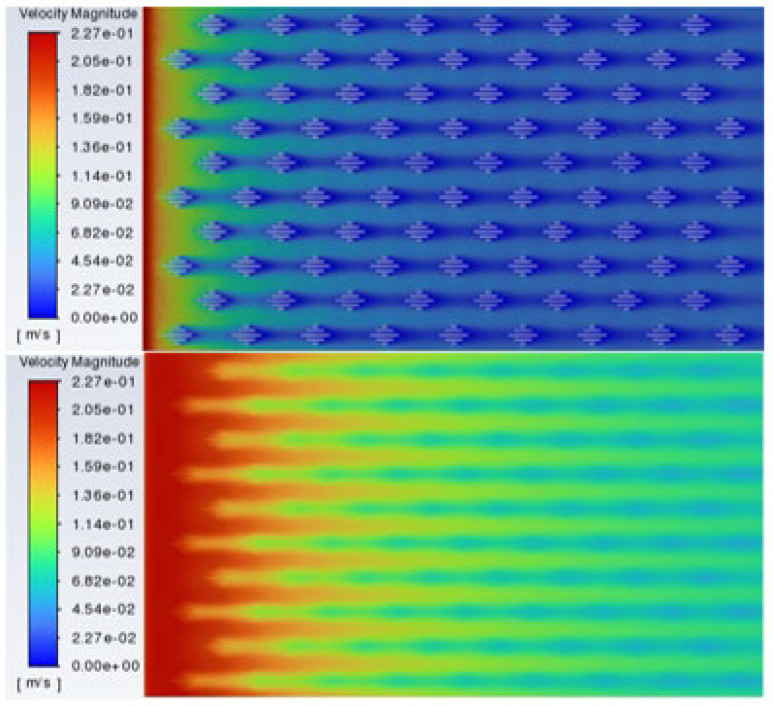
Computed velocity contour of the staggered placoid scale structure and the corresponding near-wall flow field velocity distribution.

Flow field visualization provides direct evidence for the drag reduction mechanism. Figure 10 presents the computed velocity contour of the staggered placoid scale structure and the corresponding near-wall flow field velocity distribution. The velocity map clearly reveals that higher fluid velocities and thinner boundary layers occur at the crests of the scales, while distinct low-velocity zones are formed within the grooves. This configuration establishes an ordered velocity gradient, with the low-velocity fluid in the grooves acting as an “anchored” buffer layer. This effectively reduces the near-wall velocity gradient and decreases frictional drag. The streamlines within the grooves exhibit stable recirculation patterns, which contribute to the redistribution of flow momentum and further reduce energy dissipation.

**Figure 11:**
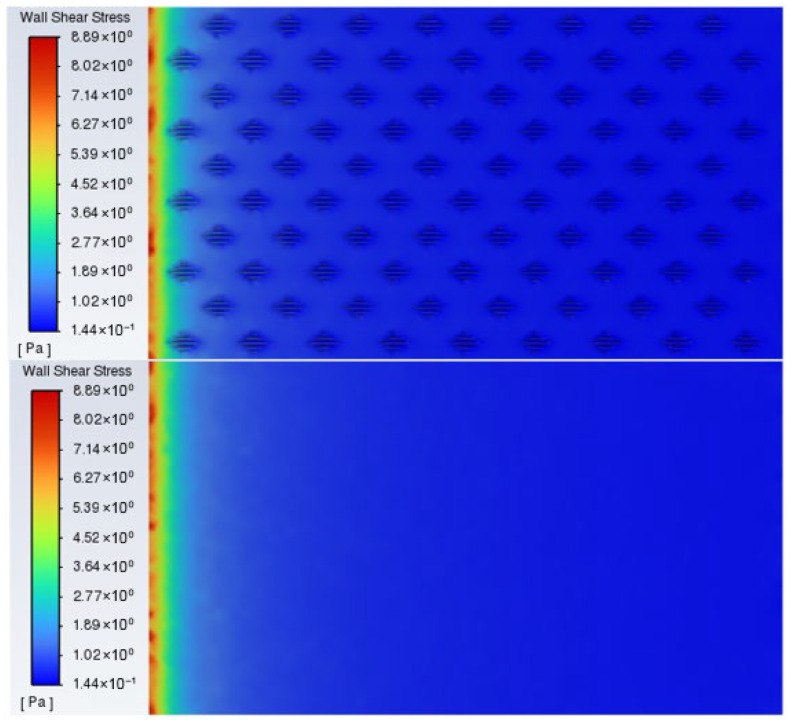
Computed wall shear stress contour of the staggered placoid scale structure and that of the smooth wall.

The wall shear stress contour map in Figure 11 presents a comparison of the shear stress distribution between the staggered placoid scale structure and a smooth surface. On the smooth surface, the shear stress distribution is relatively uniform and remains at a consistently high level. In contrast, on the biomimetic surface, the rib regions exhibit significantly lower shear stress values, appearing as darker blue areas in the contour plot. In contrast, on the biomimetic surface, the rib regions exhibit significantly lower shear stress values, appearing as darker blue areas in the contour plot, consistent with a closely related study [40]. This indicates a substantial reduction in local friction resistance. The reduction is attributed to the low-velocity fluid layer induced by the groove structures, which effectively decreases the near-wall velocity gradient and consequently lowers the local shear stress. As these low-shear regions constitute a major portion of the biomimetic surface, their cumulative contribution results in a marked reduction in overall frictional drag, thereby achieving the desired drag reduction performance. Moreover, the staggered arrangement ensures the spatial continuity of these low-shear regions, which further enhances the overall drag reduction efficiency.

Based on a comprehensive analysis of experimental results and flow field characteristics, the drag reduction mechanism of the biomimetic shark skin placoid structure originates from the regulatory effect of its unique surface topology on the near-wall laminar flow field. On one hand, the groove-rib structure establishes stable low-velocity fluid layers within the grooves, reducing the near-wall velocity gradient and consequently decreasing frictional drag. On the other hand, the staggered arrangement promotes smoother pressure distribution along the flow direction, mitigating pressure drag by reducing the pressure differential between windward and leeward surfaces. The consistency between experimental and simulated drag reduction rates, together with the flow physics revealed by the flow field images, collectively demonstrates the effectiveness and potential of this biomimetic microstructure design for low-speed underwater drag reduction applications. Future work may focus on optimizing structural parameters (such as groove width-to-depth ratio and arrangement angle) to adapt to flow fields at different Reynolds numbers and to explore the scalability of these findings to higher Reynolds number regimes.

## 4. Conclusions

This study systematically investigated the fabrication and drag reduction performance of micro grooved surfaces bioinspired by shark skin placoid scales, employing an integrated experimental and numerical simulation approach. Experimentally, a biomimetic surface featuring a staggered arrangement of placoid structures was successfully fabricated using nanosecond green laser etching and a PDMS template replication process. Morphological characterization confirmed uniform groove dimensions and the structural integrity of the fabricated surface. Quantitative assessment via sedimentation experiments demonstrated an average drag reduction rate of approximately 5.65%, preliminarily validating the effectiveness of the designed structure.

Numerical simulations further elucidated the underlying drag reduction mechanisms. Flow field analysis indicated that the groove-rib topology derived from the placoid structure effectively regulates near-wall flow: a low-velocity fluid layer formed within the grooves reduces the near-wall velocity gradient, thereby decreasing frictional drag. Additionally, stable recirculation flows develop within the grooves, contributing to momentum redistribution and reduced energy dissipation. Simultaneously, the staggered arrangement promotes a smooth pressure gradient, mitigating flow separation and consequently lowering pressure drag. The simulated drag reduction rate (5.08%) agrees well with experimental results, confirming the reliable drag reduction performance of the biomimetic structure under steady flow conditions.

In summary, this work demonstrates the distinct drag reduction effect of the designed staggered placoid biomimetic structure and provides theoretical insights through flow mechanism analysis. The 5.65% drag reduction achieved under low-speed flow conditions suggests potential applicability in underwater vehicles operating at moderate speeds, such as autonomous underwater vehicles (AUVs), underwater gliders, and ship hull sections where flow velocities are relatively low. The demonstrated PDMS-based fabrication approach also offers advantages for coating existing curved surfaces due to the material’s flexibility and conformability.

However, several limitations should be acknowledged. First, the current fabrication method is limited to small-area samples (10 mm × 10 mm); scaling to larger surfaces while maintaining structural fidelity remains challenging. Second, the durability and long-term stability of the PDMS microstructures under real marine environments—including biofouling, abrasion, and UV exposure—have not yet been evaluated. Third, the observed drag reduction is specific to the tested low-speed range (0.1–0.2 m/s); performance at higher Reynolds numbers typical of full-scale vessels requires further investigation.

Future research should focus on: (1) parametric optimization of groove dimensions (width, depth, spacing) to maximize drag reduction across different flow regimes; (2) development of scalable fabrication techniques, such as roll-to-roll nanoimprinting, for large-area surface treatment; (3) evaluation of mechanical durability and anti-biofouling properties under simulated marine conditions; and (4) exploration of hybrid surfaces combining microstructures with hydrophobic coatings to achieve synergistic drag reduction effects.

## Figures and Tables

**Table 1:** Results of drag reduction tests.

Experiment Number	Smooth Surface	Non-Smooth Surface	Drag Reduction Rate
1	2.74 s	2.59 s	5.47%
2	2.64 s	2.51 s	4.92%
3	2.76 s	2.57 s	6.88%
4	2.73 s	2.58 s	5.49%
5	2.75 s	2.60 s	5.45%

**Table 2:** Boundary condition settings for the computational domain.

Boundary Name	Location	Boundary Condition
Inlet	Inlet	Velocity Inlet (0.1–0.2 m/s)
Outlet	Outlet	Pressure Outlet
Wall-smooth	Upper surface	Wall (no-slip)
Wall-rough	Lower surface	Wall (no-slip)
Wall	Left/Right sides	Symmetry

**Table 3:** Solution settings for the computational domain.

Setting Item	Setting Value	Setting Item	Setting Value
Fluid Medium	Water	Flow Velocity Range	0.1–0.2 m/s
Pressure–Velocity Coupling	SIMPLEC	Flow Model	Laminar
Time Type	Steady	Residual Criterion	0.0001
Spatial Discretization	Second-Order Upwind	Maximum Iterations	1000

**Table 4:** Fluent simulation results (with and without pressure drag).

Velocity	0.2 (m/s)		0.15 (m/s)		0.1 (m/s)	
Smooth	0.00044332088		0.00034735183		0.00027229853	
Non-smooth (No-slip, Y-component)	0.00040298451	9.10%	0.00031689041	8.77%	0.00024997415	8.20%
Non-smooth (Free-slip, Y-component)	0.00045289249	−2.16%	0.00035394537	−1.90%	0.00027708761	−1.76%
Non-smooth (Total frictional resistance)	0.00042079808	5.08%	0.00033023672	4.93%	0.00026043641	4.36%

## Data Availability

The data presented in this study are available on request from the corresponding author.
